# Enhancing IoT Security through a Green and Sustainable Federated Learning Platform: Leveraging Efficient Encryption and the Quondam Signature Algorithm

**DOI:** 10.3390/s23198090

**Published:** 2023-09-26

**Authors:** Turki Aljrees, Ankit Kumar, Kamred Udham Singh, Teekam Singh

**Affiliations:** 1 Department College of Computer Science and Engineering, University of Hafr Al Batin, Hafar Al-Batin 39524, Saudi Arabia; tajrees@uhb.edu.sa; 2 Department of Computer Engineering & Applications, GLA University, Mathura 281406, India; iiita.ankit@gmail.com; 3 School of Computing, Graphic Era Hill University, Dehradun 248002, India; 4 Department of Computer Science and Engineering, Graphic Era Deemed to be University, Dehradun 248002, India; tsingh@ma.iitr.ac.in

**Keywords:** Internet of Things, data encryption, quondam signature algorithm (QSA), MITM, communication cost, identity-based online/offline

## Abstract

This research paper introduces a novel paradigm that synergizes innovative algorithms, namely efficient data encryption, the Quondam Signature Algorithm (QSA), and federated learning, to effectively counteract random attacks targeting Internet of Things (IoT) systems. The incorporation of federated learning not only fosters continuous learning but also upholds data privacy, bolsters security measures, and provides a robust defence mechanism against evolving threats. The Quondam Signature Algorithm (QSA) emerges as a formidable solution, adept at mitigating vulnerabilities linked to man-in-the-middle attacks. Remarkably, the QSA algorithm achieves noteworthy cost savings in IoT communication by optimizing communication bit requirements. By seamlessly integrating federated learning, IoT systems attain the ability to harmoniously aggregate and analyse data from an array of devices while zealously guarding data privacy. The decentralized approach of federated learning orchestrates local machine-learning model training on individual devices, subsequently amalgamating these models into a global one. Such a mechanism not only nurtures data privacy but also empowers the system to harness diverse data sources, enhancing its analytical capabilities. A thorough comparative analysis scrutinizes varied cost-in-communication schemes, meticulously weighing both encryption and federated learning facets. The proposed approach shines by virtue of its optimization of time complexity through the synergy of offline phase computations and online phase signature generation, hinged on an elliptic curve digital signature algorithm-based online/offline scheme. In contrast, the Slow Block Move (SBM) scheme lags behind, necessitating over 25 rounds, 1500 signature generations, and an equal number of verifications. The proposed scheme, fortified by its marriage of federated learning and efficient encryption techniques, emerges as an embodiment of improved efficiency and reduced communication costs. The culmination of this research underscores the intrinsic benefits of the proposed approach: marked reduction in communication costs, elevated analytical prowess, and heightened resilience against the spectrum of attacks that IoT systems confront.

## 1. Introduction

The Internet of Things (IoT), which makes use of small devices to monitor and manage a variety of operations, is very important to our day-to-day activities in the present day. The phrase “Internet of Things” (IoT) was first proposed by Kevin Ashton in 1999. It describes the practise of linking commonplace electronics and appliances to the internet. These globally interconnected devices amass vast troves of data and autonomously execute specific tasks, minimizing human intervention. In the contemporary cyber landscape, the IoT and its guiding principles hold sway, solidifying their dominant position. A report by Gartner [1] predicted a staggering presence of 21 billion interconnected devices by the close of 2020, underscoring the extensive potential of utilizing the Internet for multifaceted applications, ranging from smart grids to smart logistics and smart cities. However, every stride forward encounters formidable challenges. The constrained nature of IoT devices, marked by limitations in throughput, longevity, and computational capacity [2], presents novel hurdles for researchers, communication specialists, and cyber experts. Forming an efficient and sustainable ecosystem that facilitates seamless communication among these restricted devices emerges as a pressing necessity. Crafting an adept network is intricate due to the disparate standards and protocols employed by various devices. Consequently, the establishment of a heterogeneous network becomes imperative, enabling efficacious and secure communication across the entire spectrum of devices.

**Figure 1:**
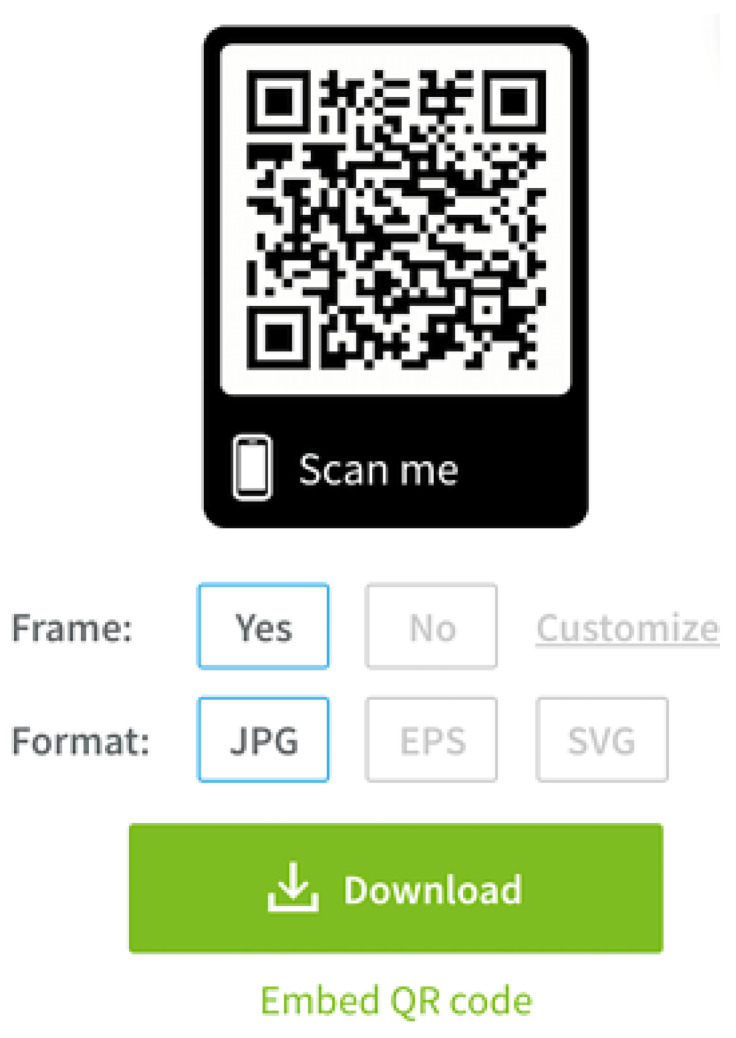
An authentication method using QR codes.

An important factor in the IoT context is security. Cybercrimes and cyberattacks have risen dramatically in frequency during the past several years [3]. Constrained gadgets have become top targets for cybercriminals because they lack strong security capabilities. Numerous instances of attackers taking control of such devices and deploying bots to launch DDoS assaults have been documented. The ecosystem of the Internet of Things (IoT), which is recognized for its gadgets with limited resources and low network throughput, runs into problems when attempting to use the same security and network protocols that are often utilized in traditional Internet systems. It is vital to continually develop and establish innovative protocols in order to respond to the specific requirements of security and communication within the IoT environment [4,5]. This is necessary in order to take into consideration issues such as low power use, limited throughput, and simpler processing. Lightweight security methods are necessary for securing the operation of apps in the IoT context. Strong authentication techniques for both devices and networks should be included in these protocols. Processes of authentication confirm the participants’ identities. Multiple credentials should be used in the process for improved security procedures to provide strong authentication. The incorporation of computationally expensive cryptographic primitives used in traditional markets is not an option for IoT businesses. Given that IoT devices are often less capable than gateway nodes, using gateways as middleware for computing raises the barrier to computational complexity [6] shown in Figure 1.

### 1.1. Federated Learning in IoT Systems

Federated Learning is a ground-breaking method that has considerable potential to improve the intelligence of Internet of Things devices as well as their level of security and privacy. The issues brought about by the dispersed nature of sensitive data in IoT systems are addressed by the fact that this technology makes collaborative machine learning possible without compromising the privacy of users or devices. When it comes to classical machine learning, training models often include the usage of centralized data stores. On the other hand, since data may be stored on a wide variety of devices, this strategy is not only impracticable but also unsafe for use in IoT systems. The problem may be solved with the help of Federated Learning, which dispenses with the need of transporting data to a centralized location by allowing the local training of machine learning models on endpoints. Each IoT node trains its model locally using the data collected by that node, and then those locally trained models are combined to produce a global model. Crucially, this aggregation process preserves the privacy of users and their sensitive data. Incorporating federated learning into IoT systems brings several benefits. Firstly, it ensures user privacy since data never leave the devices, mitigating concerns about the exposure of personal or proprietary information. This is particularly important given the sensitive nature of IoT data. Additionally, federated learning supports continuous learning and adaptation by enabling local model updates without constant communication with a central server. This flexibility is valuable in dynamic IoT environments where network connectivity may be limited. Moreover, federated learning reduces communication and bandwidth requirements, enhancing system efficiency. Rather than transmitting large volumes of data, only model updates are exchanged during the aggregation process. This reduces latency, conserves network resources, and enables real-time learning in resource-constrained IoT settings. Incorporating federated learning into IoT systems provides a powerful solution to enhance safety, privacy, and intelligence. It enables collaborative learning while preserving data privacy, supports continuous learning in dynamic environments, and improves system efficiency. As the IoT continues to expand, federated learning offers a viable approach to leverage the collective intelligence of IoT devices while ensuring the security and privacy of sensitive information.

### 1.2. Challenges and Considerations in Implementing Federated Learning

While federated learning offers numerous advantages for IoT systems, there are also several challenges and considerations to address:

- Data heterogeneity: IoT devices generate data with varying qualities, formats, and distributions. Managing this diversity while ensuring model compatibility and efficient aggregation can be challenging. Preprocessing and data-normalization techniques may be required to overcome these challenges.
- Capacity, connectivity, and power limitations: Many IoT devices have limitations in capacity, connectivity, and power when communicating with each other. It is crucial to transmit model updates efficiently and aggregate them while considering these limitations. Optimizing communication protocols and developing lightweight models suitable for IoT devices are important factors to consider.
- Security and privacy: While federated learning aims to preserve data privacy, it is essential to protect models and prevent model poisoning attacks. Robust authentication, encryption, and secure aggregation protocols need to be implemented to ensure the integrity and privacy of both models and data.
- Federated optimization: Effective optimization techniques are required to achieve convergence and generate accurate models in federated learning. Dealing with non-i.i.d. data, balancing model updates across devices, and addressing varying computational capabilities of devices are challenges to overcome.
- Device heterogeneity: IoT systems comprise devices with different processing powers and hardware features. Striking a balance between leveraging the collective intelligence of all devices and accommodating the limitations of resource-constrained devices is crucial. Methods such as model customization and adaptive learning can be explored to handle device heterogeneity.
- Bias and fairness: Federated learning may introduce biases and fairness issues when certain devices or populations are underrepresented or have imbalanced data distributions. Ensuring fairness in model training requires careful consideration and mitigation strategies to address potential biases.
- Regulatory and legal compliance: Federated learning involves handling and processing sensitive data, raising concerns about regulatory and legal compliance. Adhering to privacy regulations and implementing data protection measures is necessary to ensure compliance with applicable laws and standards.

### 1.3. Motivation

Security is of the utmost concern in the deployment of Internet of Things (IoT) systems. Protecting users and systems from cyber-attacks and crimes is a top priority. As IoT technology advances, attackers employ various methodologies to compromise user information. Therefore, there is a pressing need for a robust authentication process in network security, applicable to both small local networks and large cloud servers. The exponential growth of IoT necessitates a strong authentication technique to combat complex cyber-attacks. Furthermore, it is crucial that the authentication tool developed is unique, sophisticated, and user-friendly. Instances frequently arise where attackers gain physical access to IoT devices due to unattended users and the distributed nature of these devices across large areas. Data communication also becomes a target for intruders, particularly in wireless communication scenarios with open environments that provide opportunities for data attacks. The key components of IoT systems, the sensors, face limitations in terms of energy capabilities and processing time, making it challenging to implement complex security schemes. Because of this circumstance, there is a greater potential for unauthorized access to devices, and there are more opportunities for fraud in the authentication process. In order to find a solution to these problems, we came up with a method of authentication for smart devices that we call the Physical Unclonable Function, or PUF. The primary emphasis of their study was an investigation of the communication expenses connected to the IoT-based PUF system [7]. PUF schemes act as a catalyst for the creation of one-of-a-kind authentication protocols that have minimal overhead in terms of their communication requirements. In light of this, the present study suggests an innovative method for gaining access to data and authenticating users inside IoT devices [8]. Given the frequency of cyberattacks, it is of the utmost importance to protect the security of systems that are connected to the Internet of Things. The proposed research addresses these concerns by introducing a distinct authentication process applicable to small and large networks. By leveraging authentication schemes such as the Physical Unclonable Function (PUF), the research seeks to develop unique and cost-effective solutions for data access and authentication in IoT systems.

### 1.4. Problem Statement

In the Internet of Things (IoT), establishing robust connectivity across diverse networks and intelligent devices is of paramount importance. Nonetheless, the security of electronic apparatuses and tangible devices faces significant challenges rooted in the realms of key management, data encryption and decryption, amalgamation, and authentication. These difficulties are the result of intrinsic resource restrictions, which include issues such as restricted battery life, processor speed, and storage capacity [9,10]. This inquiry provides pioneering algorithms that have been precisely customized for the encryption and decryption of data. These algorithms revolve on the concept of using one-time accessible keys to authenticate data, which is a novel approach. The methodology posited in this discourse ensures the preservation of data integrity and bestows communication cost-effectiveness that outshines prevailing methodologies. The results underscore the significance and originality of the formulated approach, which introduces an innovative data encryption and decryption hallmark christened the “Quondam Signature Algorithm (QSA).” This hallmark serves the purpose of establishing secure exchanges between IoT applications and tangible devices. The cardinal objective of this algorithm resides in mitigating the vulnerability to man-in-the-middle (MiM) attacks.

### 1.5. Major Contribution

The major contributions of this research paper can be summarized as follows:

1. The paper introduces inventive algorithms that merge effective data encryption, the Quondam Signature Algorithm (QSA), and federated learning. By incorporating federated learning, the paper facilitates continual learning while upholding data privacy, bolstering security measures, and shielding against evolving threats in IoT systems.
2. The research puts forth the Quondam Signature Algorithm (QSA) as a remedy to counteract vulnerabilities to man-in-the-middle attacks within IoT systems. The QSA algorithm curtails communication bit requisites, leading to cost savings in IoT communication and enhancing overall system security.
3. Through the assimilation of federated learning, the proposed approach enables IoT systems to amass and analyse data from multiple devices while upholding privacy. The system harnesses diverse data sources without jeopardizing data privacy by training machine learning models locally on each device and then consolidating them to craft a global model.
4. The research juxtaposes distinct cost-in-communication strategies, taking into account both encryption and federated learning facets. The proposed approach streamlines time complexity through computations in the offline phase and signature generation in the online phase, employing an online/offline signature scheme based on elliptic curve digital signatures.
5. Among the assessed schemes, the proposed strategy, which fuses federated learning and efficient encryption methods, demonstrates heightened efficiency and diminished communication costs. It outperforms the Slow Block Move (SBM) scheme, diminishing the number of rounds, signature generations, and verifications necessary.

The paper is structured as described below. The introduction offers a summary of the goals of the study and emphasizes the need of developing original algorithms in order to defend Internet of Things systems from random assaults. Section 2, as the literature review, explores related works on data encryption, federated learning, and signature algorithms. Section 3, as the methodology section, presents the integration of efficient data encryption, the Quondam Signature Algorithm (QSA), and federated learning in addressing IoT security challenges. The comparative analysis evaluates different cost-in-communication schemes, considering encryption and federated learning aspects. The results and discussion, as Section 4, presents the findings, including the improved efficiency and reduced communication costs of the proposed approach. The conclusion summarizes the contributions and suggests future research directions.

## 2. Literature Survey

The Internet of Things (IoT) comprises various electrical parts, such as sensors, actuators, and software. It stands for a heterogeneous and embedded network of interconnected “things” that interact and share data online. IoT devices include sensors and need computing power to function well in a variety of settings, including industrial warehouses, woods, and agricultural fields for crop monitoring. Smart grids, smart parking, and smart healthcare are examples of common IoT applications. In a 2013 analysis, the International Data Corporation predicted that by 2020, 41 billion IoT devices would be connected to the network system, with a market growth expected to be 8.9 trillion dollars [11]. IoT and conventional markets differ significantly in that there is no direct human involvement. IoT devices manage duties including the gathering and analysis of data on individual behavioural patterns [12]. These programs provide useful services that are very beneficial to human existence. However, maintaining individual security and privacy protection in IoT applications comes at a significant expense. IoT manufacturers have frequently failed to install and deploy reliable, secure systems at the device level. As a result of the numerous susceptible gadgets connected to the internet, which increases system complexity, security experts have issued warnings about possible threats [13].

IoT devices face substantial difficulties related to privacy and security since they usher in a new phase of disruption that can potentially violate customers’ privacy to varied degrees. This case arises due to the high potential of IoT devices. Such devices not only attack and collect the personal information of users, such as their names, residential addresses, date of birth, phone numbers, etc., but also trace their day-to-day activities and movements, such as when they went for a vacation, where they were staying, what their food was, etc. Due to a continuous spate of significant data breaches, users have legitimate and well-founded worries regarding storing large amounts of personal data in databases connected to public or public cloud platforms [14]. 

Numerous articles, research papers, and surveys have been published addressing the privacy and security issues of IoT, highlighting the various challenges faced by users. Additionally, a comprehensive analysis of existing approaches has been carried out, classifying them into four common IoT communication protocols: application, network, Medium Access Control (MAC), and physical [15]. Researchers [16] have also mentioned current security trade-offs and privacy restrictions as significant research roadblocks in the area. They categorized the identification of potential security issues into several areas, including policy enforcement, secure middleware, trust, access control, authentication, confidentiality, mobile security, and privacy.

To minimize data processing latency by putting devices closer, Cui et al. devised a unique encryption approach using a proxy-assisted attribute for edge-level security [17]. In a similar spirit, to reduce the running power consumption of IoT devices, the authors of [18] suggested an authentication system based on social networking. For each IoT application, this protocol establishes customized multi-security levels of authentication.

Through the use of a granular bulk operations algorithm, Zhou et al. created an effective access-control system for interactive IoT devices [19]. A tracking attacker model that is applicable to both centralized and distributed IoT infrastructures was suggested by the authors of another piece of research that looked at the proliferation of IoT systems [20]. They also invented a symmetric key cryptosystem, which makes use of a single, user-only key for both encryption and decryption. In addition, L2D-CASK, a technique of encryption was introduced for protecting sensor data in FPGA domains [21]. A hybrid CA rule vector with a key length of 512 bits was used in this method, along with an XOR operation that can take two or three inputs at the same time. This strategy successfully handles the memory limitations of sensor nodes, guaranteeing optimum performance without jeopardizing the computational and randomization challenges of encryption. By using the innate abilities of cellular automata (CA), it produces chaotic sequences [22,23]. The plan exhibits efficiency in terms of randomness, which qualifies it as a system for comparable encryption, like DES and 3-DES. The suggested method improves system robustness by choosing key configurations at random and setting the number of encryption and decryption iterations.

The article [24] delves into the realm of IoT networks and explores how the integration of blockchain technology can enhance both privacy and security. By leveraging blockchain’s inherent decentralized and tamper-resistant attributes, the study proposes a method to safeguard sensitive data and secure communications in IoT environments. The article’s contribution lies in its innovative approach to addressing the challenges posed by privacy breaches and security vulnerabilities within IoT systems.

The article [25] discusses a lightweight and safe protocol that intends to better the overall security of IoT networks. The essay focuses on the Internet of Things (IoT); hence, the protocol’s main focus is on the IoT. By combining encryption techniques and efficient communication protocols, the study presents a method that safeguards data integrity and protects against unauthorized access. The article’s significance lies in its emphasis on achieving a balance between security and resource efficiency, catering to the constraints of IoT devices.

Exploring the security landscape of IoT networks, the article [26] proposes an efficient security mechanism that combines blockchain technology and machine learning techniques. By employing blockchain’s immutability and machine learning’s adaptive capabilities, the study offers a method to identify and mitigate security threats in real-time. The article’s contribution is in its innovative integration of blockchain and machine learning to enhance the resilience of IoT systems against evolving cyber threats.

Establishing links between people and a network of ubiquitous, linked smart items is the fundamental goal of the Internet of Things (IoT). As a result of developments in unique Internet approaches, wireless communication technologies, and cost-effective micro sensors, this field has garnered a significant amount of interest in recent years. The methodologies of the Internet of Things have found use in a variety of disciplines, including medicine and healthcare, the monitoring of the environment, logistics, intelligent homes and buildings, and intelligent transportation systems. One characteristic that deserves special attention is the incorporation of context awareness into IoT systems. In the field of accumulating sensory data, the geographical information is, likewise, of great relevance [27]. Engineers and academics have made significant headway in developing more sophisticated prototypes over the course of the last twenty years. These prototypes include both system designs and outputs associated with localization and positioning modules for sensor nodes in the Internet of Things (IoT). Examples of Internet of Things (IoT) systems include configurations in which receivers for Bluetooth Low Energy (BLE), Wireless Local Area Networks (WLAN), and Global Navigation Satellite System (GNSS) are incorporated in a single device. In situations such as the one with Telit [28], one can see this integration in action. In addition, alternative approaches like CRYSTAL [29], Carriots [30], Sierra Wireless [31], and SOFIA [32] have surfaced in recent years to assist in the development of Internet of Things (IoT) systems that have the capacity to function locally.

### 2.1. Classification of Attacks in the IoT

Security is a critical issue in IoT systems that must be addressed since it is one of the biggest worries. A high degree of security must be maintained, given the requirement for effective data exchange to defend against various cyber-attacks. Assaults, including Sybil assaults, eavesdropping, message alteration, traffic analysis, and Denial of Service (DoS) attacks, offer serious risks to people and organizations, giving attackers access to private data and the chance to profit financially [33]. The IoT ecosystem’s exponential expansion has attracted more cyber attackers, who use trickier techniques to get past security barriers [33,34]. As a result, protecting IoT systems becomes more difficult and calls for ongoing attention as well as the development of strong security measures to combat these changing threats.

### 2.2. Securing the Internet of Things (IoT) through Cryptographic Techniques

In this section, we explore cryptographic techniques to ensure secure localization and protect location information in the context of the IoT. Due to the lack of well-defined standards for safe IoT localization, current techniques focus mostly on establishing applications within IoT frameworks, like RFID or WSN for military services. IoT devices’ heterogeneous design, which includes sensitive location data and worldwide connectivity, creates a variety of security risks and new user issues. Therefore, it is crucial to address these challenges by leveraging cryptographic methods to safeguard location information and enhance the security of IoT systems shown in Table 1. 

It is important for the people engaged to share a secret key in order to create safe communication. There are two methods to do this:

- By making use of a pre-shared secret that is hardcoded into the gadgets before they are put into service.
- By creating and dispersing the shared secret key via a key exchange protocol [2].

It is worth noting that public key cryptography, while more secure, can be more expensive to implement compared to other methods.

### 2.3. Encryption and Decryption Techniques

To protect information privacy and guarantee data security, a variety of encryption and decryption techniques are used. Conventional technologies, however, frequently rely on insufficient resources that have constrained power and processing times. As a result, these technologies are not appropriate for use in devices that have limited resources, such as those that are found in the Internet of Things scenario. In response to this problem, lightweight cryptographic algorithms that are tailored specifically to the requirements of such devices, in terms of their size and processing power, have been developed. Among the different cipher blocks, block ciphers have demonstrated favourable performances [36]. In the realm of resource-constrained devices, Crypton serves as a 64-bit lightweight cipher suitable for applications in Wireless Sensor Networks (WSN) and RFID [37]. Additionally, Crypton, as a 128-bit cipher, offers alternative options to AES with improved system performance [38]. In terms of resilience against attacks, secret keys such as Hummingbird-1 and Hummingbird-2, with lengths of 128 and 256 bits, respectively, have been proposed [39]. These cryptographic techniques provide enhanced security while addressing the specific constraints of resource-constrained devices.

### 2.4. Available Mobile Signature Solution

**Figure 2:**
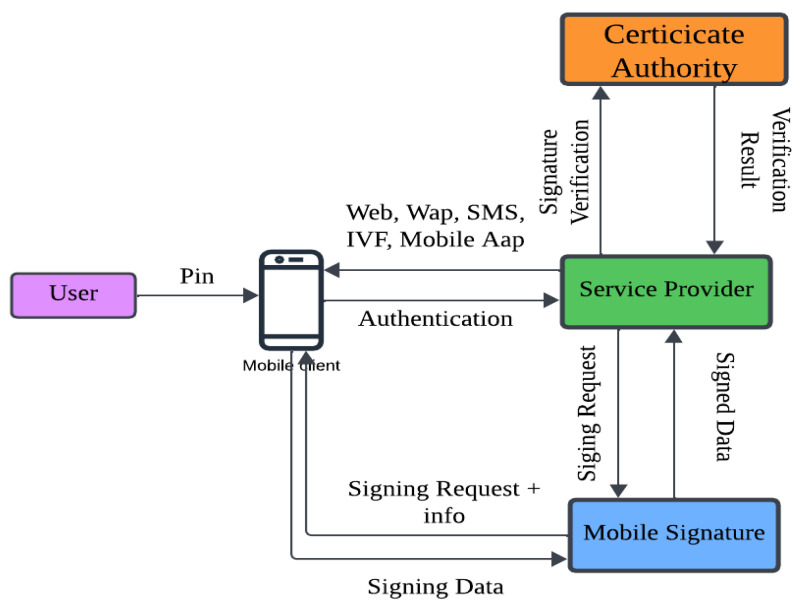
The user authentication process utilizes the existing mobile signature solution that is currently available for the operation.

In recent times, novel methodologies for data encryption/decryption have emerged as solutions to counter various attacks. One solution to address these challenges is the utilization of mobile signatures, which facilitate the creation of qualified digital certificates for user authentication. These certifications are compliant with ETSI standards and are issued by authorised Certificate Authorities. ETSI is an abbreviation for the European Telecommunications Standards Institute. ETSI has only recently made available for public evaluation a draught standard that outlines a framework for verifying and standardising advanced electronic signatures (AdES) in mobile settings [40]. As an option to improve one’s level of privacy, EAL+-certified SIM cards are now accessible to the general public. A user’s subscription to the service triggers the activation of a certificate that is sent over the air. The secret key is safely maintained on the SIM card, while the public key is made accessible in a directory. Each certificate is made up of distinct public and private keys that are kept in different locations. When a user attempts to obtain access to a service, the service will first send a request to the user to provide authentication information as well as a signature [41]. A One-Time Password (OTP) or Personal Identification Number (PIN) is presented by the user’s device before gaining access to the signature. After that, the mobile operator sends this signature to the service provider, which then enables safe and authorised access to the service that the user desires. The signature is verified by the supplier of the service, who then gives access to the service that was requested. The process of using mobile signatures for user authentication is broken down into steps, which are shown in Figure 2.

### 2.5. Man-in-the-Middle Attack [42]

The phrase “Man-In-The-Middle”, which is sometimes abbreviated as MitM, MITMA, MITM, or MiM in academic writing, refers to an assault in which an intermediate party gets covert control over the communication channel between two or more organisations. This attack provides the methods to the attacker for replacing, changing, modifying, or intercepting the communication traffic of the targeted victim. The victim believes that the communication channel is secured due to the unawareness of the MITM attack [43]. These attacks permit the attacker to send, receive, change, and intercept information, without the knowledge of an outside party, which was never meant to be for them. These can be used to invoke attacks such as session hijacking, port stealing, DNS spoofing, and distributed denial of service (DDOS) attacks. MITM can steal local FTP IDs; stealing the Password and ID of the online user has severe consequences. These attacks depend on the user’s identity and can be passive or active. The attacker’s presence is not detected in a passive attack. 

**Figure 3:**
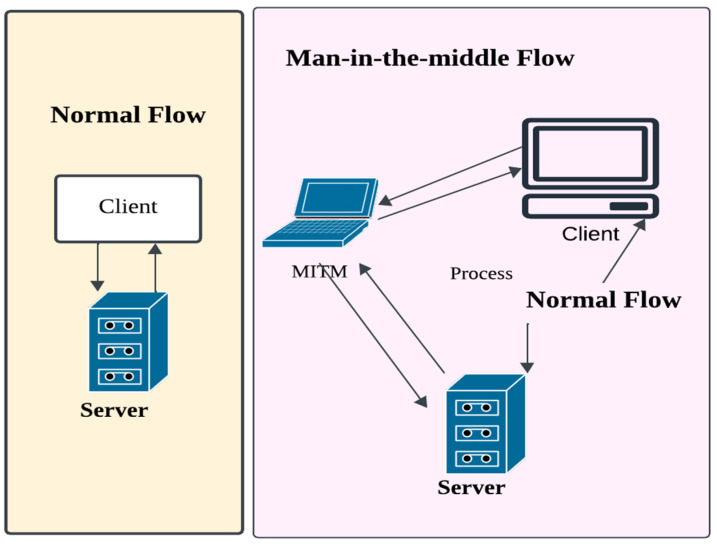
Differentiation between the flow in the MITM attack vs. data in normal flow.

MITM attacks can allow the attackers to insert themselves between two communicating terminals without getting detected. These kinds of attacks consistently lack certain features, which makes them rare on the wired internet. There is the occurrence of contradictions in the situation for wireless connection. Depending on the nature of the protocol as a wireless link layer, the attacker can effortlessly insert user information. Figure 3 illustrates the difference between the flow of data in MITM and data in regular communication, or the normal flow of information. In general, the two parties can communicate with each other in normal flow, i.e., the server and client, without any mediation or intrusion of any MITM. While the communication is occurring between the man in the middle and the attacker in the MITM flow.

Therefore, the server was facing the victim as the spoof of the attacker. Attackers can influence the availability, integrity, and confidentiality of the information. A MITM attack is prone to the storage of cryptocurrencies. Once, there was a safer method to store cryptocurrencies considered by researchers. In that method, the MITM attack [44] exposed the vulnerability of ledger hardware wallets. This attack permits a cybercriminal to create a duplicate or clone address of the cryptocurrency by showing it to the customer and utilizing the original address to deliver it to the wallet. Later, the cybercriminal delivers the cryptocurrency to a fraudulent address instead of the user’s wallet. The results also affect the user’s system, as the addition of malware to infect the victim’s computer leads to accommodate the MITM attack.

### 2.6. Authentication Scheme-Based Physical Unclonable Function for IoT-Featured Smart Devices [45]

Today, information technology and its advancement are not only restricted to the zone of making communication between people. Intelligent technologies/smart innovations with an enhanced automation process warrant the connectivity of IoT in communication between persons. It creates a close platform for the development of a mature link between things, people and objects through network and sensor technology. The IoT has received worldwide and widespread attention. In recent years, we have witnessed the applications of IoT in the sectors of environment protection, healthcare, agriculture, smart cities/homes, smart grids, and smart things in intelligent transportation. However, severe challenges and issues are present for physical objects such as key management, authentication, and integration. Internet in the smart world faces the rapid growth of ubiquitous connection between things and people. It also brings more security challenges, as it will undoubtedly in the future.

Secure communication between two parties allows verification through the authentication of the user credentials. One-way hash function auxiliary information uses authentication schemes to perform random key pre-distribution, pre-shared, and existing asymmetric functions. Asymmetric and symmetric ciphers include identity-based authentication that may be utilized for authentication as well as biological characteristics. Such unique binding properties can achieve biological characteristics such as stability, naturality, non-repudiation, tamper-resistance, and other characteristics. In the field of security certification, user biometrics becomes a trending topic, using the face, iris, retina, DNA, fingerprints, and other authentication. Function-based encryption involves hash-based authentication functions or message authentication codes.

The Physical Unclonable Function (PUF) refers to a response to a physical entity as an output and input challenge. This response indicates misalignment due to the inherent structure of physical entities and relates to the random variations in physical structure. Each type of material requires a different design, as some existing PUFs only support the access control or authentication of the PUF. Only recently has PUF become a physical technology, and research remains in its infancy because of the effectiveness of measurements on process and manufacture of process variation in order the influence of random factors. Therefore, it produces a stimulus–response having non-cloning, non-estimated, random, and unpredictable features. The PUF is widely used in security device authentication, the protection of intellectual property and key generation such as in IPTV, accessibility of smart home appliances, product verification, and accessing other multimedia services. Currently, PUF research consists of studies investigating on the design of integrated circuits (IC) as ring oscillator PUF, SRAM PUF, and arbiter PUF. Another model is available as a PUF-based Authentication Scheme (PAS). In a PAS, users initially explore the registration process for the accessibility of smart homes through smart devices, and the requests get initiated to the requester device. Later in the registration process, if the system cannot find the identity of the device from the registration id of fingerprint, then the gateway can save it. It delivers the issue to receive a valid response from the device to authenticate it. Some of the smart devices, such as wearables and cell phones, act as requesting devices for maintaining a session key. It is further utilized for secure command execution during the next protocol.

### 2.7. Background of PUF Schemes

This section explains the PUF-based schemes with the exploration of other authentication schemes. There is a presence of numerous schemes based on fuzzy extraction algorithms that depend on the PUF methodology. In addition, PUF-based mutual authentication schemes explain the architecture designs for future IoT systems. The IoT is massively increasing in the number of connected devices and has been rapidly growing in recent years to produce new security and other challenges in the IoT. Systems based on radio-frequency identification (RFID) are designed to manage the authenticity of devices. These systems particularly work for short-range wireless radio communication and play a vital role in utilizing the readers and tags. The work of tags is to achieve power from a reader for obtaining the credentials from memory and accessing their identity. The user device operates the scheme with secured cryptographic operations that hinders the traditional complex techniques. Similarly, these tags are being utilized by new solutions to enhance their authenticity, by including the PUF between the reader and user for secure communication. Attacks show vulnerability in resulting labels, including in identity forgery, tampering tag data tracking, and location privacy exposure. The PUF ensures randomness for providing security by addressing its issues with the utilization of the hardware characteristics of its resource-constrained devices. P. Tuyls et al. provided offline PUF authentication by suggesting digital signature schemes with a combined identification process. In the certification process, an embedded PUF-enabled device considers the tag as RFID. The label produces auxiliary data and PUF’s key identity. Later, a PUF attached with identity performs the verification process. Researchers have proved a reduction in the resistance to attacks using such a scheme [45]. Kulseng suggested a PUF combined with a linear feedback shift register and RFID system. The response of the PUF is used for generating a random key in the linear feedback shift register and to tag identity authentication for providing secure communication [46].

Authentication schemes also feature biological properties in PUF. These can attain the merits of randomness and uniqueness of microstructure-based electronic components that cannot be damaged easily. The change in its stimulus–response behaviour occurs only if the attacker is trying to invade a PUF device, leading to the incentive response behaviour of each PUF. There is a solution to these problems through mutual authentication between entities using the challenge response characteristics of the PUF. Another merit is that the same challenge response can provide unpredictability and randomness in the PUF scheme. Such schemes can also be utilized for producing the keys for data decryption and encryption. The origination of PUF occurred as a physical one-way function [47]. However, in past years, PUF architectures can be developed and classified as non-silicon and silicon-based PUFs. Non-silicon PUF is mostly greater in size and produces a large number of delays in the system. On the other hand, silicon PUFs have been widely adopted and utilized in a number of applications. It is fabricated and integrated as silicon [48]-based circuits and considered as a class of PUF, while its subclass is considered as an electronic PUF. 

An alternative way exists in PUF schemes, such as controlled physical random functions (CPUFs) in place of the storage of secret keys. It presents the integration of additional circuits using hash functions to strengthen the chain of secrets used for challenge-response pairs [49]. The PUF property makes the characterization in inherent PUF [50], as it consists of a null change in the human features for regulating the behaviour of the challenge-response. PUF-based logic reconstruction was developed to dynamically alter the response behaviour in the incentives of PUF [51]. At the same instant, material production is based on time, and different non-silicon PUFs can involve a paper PUF and CD PUF. The basic principle is to control an object or entity’s inherent random nature and facilitate the realization of PUF expression. Due to strong randomness, PUF generates a set of unique keys that is applicable to IoT applications [52]. Cryptographic functions use biometric data inputs for extracting the fuzzy functions [53] utilized for encryption, authentication, and decryption. This extraction process, based on the fuzzy function, consist of two main elements: a random extractor (Random Extractor, Ext) and schematic security (Secure Sketch, SS). An error-correction mechanism, depending on the embedded data to make a schematic reproducible with a security key goal, receives the same data each time. Another element a random extractor key targets is the production of unpredictability and randomness in the data sets. By extracting the compression functions, it attains the maximum and minimum entropies at the output values of the PUF such as HMAC, hash function, SHA-1, etc. Two stages are featured, reconstruction and registration, for the implementations of specific functions. Key generation is the registration phase, in which function Gen () is generated and used for registration. Another phase is to generate the key again with the help of the reconstruction phase function to reproduce Rep (). The generated function Gen () is a response to the auxiliary data key, the standard output, and the input, while the error of the response is shown as Rep () for the output key and auxiliary data input. Another methodology is the combination of error correction with cryptographic operations [54]. The database contains the program designs for specific task implementation, such as the extraction code to blur the offset structure for this scenario. It initiates with the consideration of large-code word space with the selection of a random code word, C, where r is in order to ensure security. Later, r ⊕ c makes the calculations for auxiliary data, obtained as H.

The IoT provides security in consideration of distributed host identity-based protocols (HIP). Due to extensive operations, it causes communication and computation overheads. Reduction in its cost of computation has been achieved in several studies [55], while other suggested methodologies still face a high cost of communication, which is further lessened with the help of the proposed scheme. The fuzzy extractor depends on the extraction algorithm [56], the soft decision-based fuzzy algorithm for extraction [57] on-checksum [58], and pattern matching [59] techniques. The fuzzy checksum-based extraction algorithm makes the calculations for restoring the standard data after achieving the value from the error vector. Fuzzy bit soft decisions are being used as trust extraction algorithms for achieving security solutions as part of the auxiliary data. This results in the minimum entropy loss with a reduction in the error-correction process. The performance of the error-correction process is improved with the selection of the optimal decoding technique. To obtain the true value of the PUF response, there is the consideration of input coming from the parity index of the fuzzy extraction algorithm. The index function maps the functions for the reconstruction of the key. During the preparation phase, the generation of n bits occurs from the PUF response after the selection of an index substring matching pattern with a random number of a certain length. The regeneration phase carries the non-volatile memory for storage of the index substring matching pattern. It authenticates the device by performing the matches between the substring and the PUF output pattern. Excitation sets generate the keys where the generated patterns do not require error-correction algorithms. 

In the DTLS scheme, RSA-based certificates as certificate-based schemes perform authentication by involving two-phase authentication, as the implicit certificate (ICRT) and x.509 certificate [60] are utilized [61]. The ubiquitous IoT (U2IoT) and unit as an architecture for future networking (U2IoT) [62] are growing towards worldwide networking applications from a single crosscutting area. Some of the security issues need mandatory addressing towards the future of things through the U2IoT architecture as a safety solution. Exploration of U2IoT requires security with the concept of the Object Life Cycle (OLC). The smart home scenario explores authentic phases and registration through the combination of a mutual authentication protocol (MAP) and OLC-based PUF [63]. Compared with this approach, a secured command execution protocol along with an authentication scheme and efficient registration is further suggested. Some projects consider the Future Internet Architectures (FIA) [64], SENSAI [65], and PECS [66].

### 2.8. Identification and Security IOT Challenges [67]

IoT features with an identification scheme must be able to encompass communication objects (e.g., tags, sensors) as local topologies, with the existence of multiple identification systems during the compatible maintenance of Internet legacy. It must sustain multi-homing and mobility nodes. Apart from these two features, other rationales are often presented for a locator in the legacy Internet and decoupling identifier. The IoT presents certain scenarios that require resilience (e.g., supports the sleepy nodes from a transparent system through a gateway masquerading as them) and aggregation (e.g., a common identifier used as multiple nodes under a reverse multicast technique). Later, strengthening occurs with these functionalities on the need for locator/identifier split. The underlying identification technique must be skilled for bootstrapping, which creates a safe context between two IoT nodes because of the deficiency of a global security infrastructure. Before establishing a secured context, through their respective identifiers, there must be no expectation to attain more knowledge of each other. Therefore, an identification protocol is required for delivering the key agreement, identifier ownership, and the locator/identifier split.

#### 2.8.1. Paradigm for Locator/Identifier Split

The underlying concept operates on a fundamentally different principle, using distinct values to determine the appropriate routing and destination for a data unit, and ensuring it is sent to the intended location. At the same time, it consists of two successive distinct nodes where a single node can alter its location. Additionally, the data unit provided by an application is only concerned with identifying the recipient’s identity, as opposed to the intermediary nodes and routing components in the source, which are entirely focused on pinpointing the recipient’s location. However, the two concepts become mixed with legacy Internet, involving the addresses of IPs for both naming (identifier role) and addressing (locator role). In IP-based systems, the separation of the locator and identifier introduces a range of protocols that researchers have examined in their studies, often by implementing an identification layer above the IP layer. 

The identities of their respective identifiers get hidden from each other during the establishment of a secure connection between two IoT nodes, which has already been illustrated in this section under the introductory text. Even without the presence of a global Public Key Infrastructure (PKI), nodes must take on responsibility in the case of mutual authentication. To use these identities as security enablers, IoT nodes must be able to trust them. IoT anticipates the introduction of a secure resolution mechanism with IDs to fulfil this requirement. A node A seeks to connect with a node B to resolve, discover, and search the requested peer as part of the IoT resolution procedure. For instance, node A may use a sophisticated resolution system that offers a set of qualities to ask for “the closest milk pack” or “a nearby temperature sensor”. 

In contrast to the old Internet DNS, the IoT’s enhanced resolution mechanism acts differently. By offering trustworthy ideals in a trustworthy manner, it must build trust. A cross-check with the traditional Internet DNS should be performed to confirm the validity of a value supplied by the IoT resolution system. This may be conducted by checking the resolved node’s certificate and content. Node A must believe that node B can provide the desired service or feature and that the identifier of node B acquired from the IoT advanced resolution system is accurate, because cross-checking procedures might not be easily accessible.

#### 2.8.2. Key Agreement and Identifier Ownership 

The identification having been established by node A, which may be reliably considered to belong to node B, is retrieved by that node. Nevertheless, it is necessary to provide an explanation of the protection measures associated with this identity so that only B will be able to use it. In the event that this is not the case, it will be simple for adversaries to fake a legitimate identification, impersonate node B, and trick node A. In order to find a solution to this problem, the idea of a “secure identifier” comes into play. A secure identification cannot be faked and can only be used by the person who is legally entitled to use it. This identifier has a one-of-a-kind association with a public key, and the genuine owner is the only one who is privy to the private key that corresponds to it. As a result, providing evidence that an individual is the owner of a certain identifier is the same as providing evidence that they are the owner of a pair of private and public keys.

Within the framework of the setup of a secure connection for both nodes, mutual authentication is necessary as a prerequisite. This assures that the secret key will be shared with the appropriate peer when it is established. This insurance is very necessary to guard against man-in-the-middle (MitM) attacks so that you can stay safe. Because of this, it becomes specialized for a number of different Authenticated Key Exchange (AKE) protocols. In the realm of the Internet of Things, AKE protocols need to be used as a leverage point to protect identifiers on nodes.

#### 2.8.3. Host Identity Protocol (HIP) Rationale

A paradigm-based locator/identifier split benefits the Host Identity Protocol (HIP, [67]). An additional layer of HIP has been suggested to be placed on the top of the IP layer. A safe identification of the sender and recipient of a data unit using Host Identifiers (HIs) is made possible by the addition of cryptographic protection in the form of the Host Identity Protocol (HIP) layer. To avoid node impersonation, HIP uses a a secure Base Exchange (BEX) method. The HIP initiator and responder decide on a shared secret before beginning the BEX protocol. Therefore, HIP uses IPsec security to provide a secure session and prevent unauthorized access. The conversing peers are the ones that have completed the BEX procedure. HIP satisfies the criteria by addressing the key agreement, identifier ownership, and the locator/identifier split.

The only other Internet-identifying methods that provide cryptographic security for identifiers are SHIM6 and HIP. HIP, however, stands out as the more developed and practical Internet of Things (IoT) solution in actual use. The HIP is the recommended option for IoT applications due to its capability to extract a shared secret in an easy-to-use and safe way, support for interoperability protocols, and accessibility on a variety of devices.

#### 2.8.4. HIP Base Exchange (BEX)

**Figure 4:**
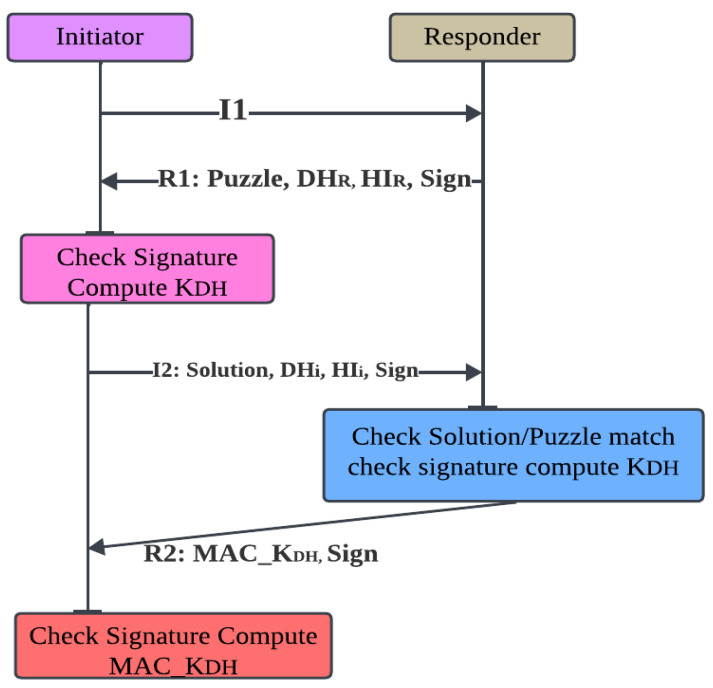
HIP Base Exchange (BEX).

The exchange procedure that the initiator delivers the information I1 to start the process is shown in Figure 4. A puzzle, a signature (for node authentication), the responder’s public key (Host Identifier), and its Diffie–Hellman (DH) public key are all included in the packet R1 that is sent after receiving I1. The responder then waits for the initiator to reply while still being stateless. The puzzle’s solution, a signature, the initiator’s public key (Host Identifier), and its own Diffie–Hellman public key are all included in the initiator’s response I2 packet. The responder verifies the answer to the problem before calculating the Diffie–Hellman session key. The final packet, R2, which completes the sending process, signals the completion of the exchange. By utilizing the created DH key to compute the exchange (signed) MAC, the initiator verifies the key.

The responder and initiator sides of the HIP Base Exchange both use complex cryptographic calculations. The calculation of two modular exponentiations, which is necessary for configuring Diffie–Hellman and producing the accompanying public keys [67,68], is the most difficult aspect of these calculations. Additionally, activities like R1, I2, and R2 that are engaged in message verification and signature calculations are still important and cannot be ignored for a resource-constrained node like R1.

#### 2.8.5. Lighter HIP Declinations

This investigation has proposed two modifications aimed at reducing the computational burden of the HIP Base exchange to make the protocol lighter.

- The HIP Diet Exchange (DEX) protocol makes use of the public value in the form of a long-term Elliptic Curve Diffie–Hellman (ECDH), such as the host identifier. Since the Diffie–Hellman algorithm itself acts as the host identifier, DEX does not need a separate public value for authentication like conventional asymmetric encryption requires. Using the exchange procedure to gather adequate knowledge of the DH key, this method validates a node’s validity. The final secret is obtained in this manner by exchanging two random seeds, x, and y, using the DH key. DEX intends to reduce the cost of producing temporary public keys, which is normally necessary for the final DH key computation in protocols like HIP BEX, by using long-term public Diffie–Hellman values for a single computation. The fact is that no other operation for asymmetric cryptography uses the Elliptic Curve Diffie–Hellman to make the key exchange lighter. A high-resource-constrained node supports the key exchange based on ECDH that is too heavy.
- Lightweight HIP (LHIP) [69] keeps the same message syntax, which is a much more radical approach like HIP BEX. However, this process does not utilize any of the security mechanisms of the HIP BEX for compatibility reasons. After the exchange, no secure IPsec tunnel is created, no RSA procedures are carried out, and no Diffie–Hellman keys are calculated. Instead, a basic amount of security is attained by employing hash chains to cryptographically tie subsequent messages. Significantly more emphasis is placed on energy saving than security in the Lightweight Host Identity Protocol (LHIP). This supports the integration of supporting node mobility as HIP control messages through a hash chains mechanism, which results in a low security level. Later, it warrants a non-hijacked ongoing session (temporal separation property) without providing the authentication to the strong node. Moreover, there is the absence of key exchange procedures, resulting in unprotected HIP data messages [70].

## 3. Proposed Methodology

This research introduces a novel encryption/decryption algorithm aimed at mitigating attacks on IoT systems. 

### 3.1. Integration of Federated Learning with Encryption and Communication

The integration of federated learning with encryption and communication in IoT systems offers a powerful approach to enhancing data privacy and security. By leveraging federated learning, machine learning models can be trained locally on IoT devices, eliminating the need to expose sensitive data over the internet. This distributed approach reduces the risk of unauthorized access and hacking attempts. To further strengthen security, encryption methods can be applied to protect the connections between devices and the central server. Secure communication protocols, such as Transport Layer Security (TLS), can be implemented to encrypt data during transmission, ensuring protection against interception and data theft.

Data encryption techniques, like the Advanced Encryption Standard (AES), can be utilized to safeguard stored data on IoT devices. This ensures that the encrypted data remain inaccessible even if a device is compromised without the proper decryption keys. The combination of federated learning, encryption, and secure communication mechanisms significantly enhances data privacy and security in IoT systems. Collaborative learning is made possible without compromising the confidentiality of sensitive information. The integration of encryption techniques and secure communication protocols safeguards data during transit and at rest. However, it is crucial to strike a balance between security and system efficiency when implementing encryption and communication protocols. It is essential to take into consideration the computational complexity of encryption methods and the way in which these methods impact the functioning of Internet of Things devices that have restricted resources. It is feasible to maintain stringent security measures without placing an unnecessary load on the system’s resources if the appropriate encryption techniques and communication protocols are used. Integrating federated learning with encryption and communication mechanisms in IoT systems provides a robust solution for ensuring data privacy and security. By combining the benefits of federated learning with secure communication protocols and encryption techniques, the system can achieve enhanced privacy, secure model aggregation, and protection against unauthorized access.

### 3.2. Federated Learning for Enhancing Data Privacy in IoT Systems

Federated learning holds tremendous potential in enhancing data privacy within IoT systems. Given the sensitive nature of IoT-generated data, safeguarding its confidentiality is paramount. Through federated learning, the training of machine learning models on IoT devices can now be achieved in a decentralized and secure manner. With federated learning, data remains localized on individual devices, and only the latest model updates are exchanged. This eliminates the need for transmitting sensitive information to a centralized server, mitigating potential security risks. As a result, user privacy is safeguarded, as data samples never leave the confines of the device.

A significant advantage of federated learning is the ability to aggregate models while preserving user privacy. Instead of transmitting data to a central server, devices locally gather and combine model modifications. This collaborative approach enables leveraging the vast collection of IoT data without compromising user privacy rights.

To reinforce the protection of user information, encryption techniques can be applied to the communication between endpoints and the central server. Secure communication protocols such as Transport Layer Security (TLS) can encrypt data in transit, rendering it indecipherable to unauthorized parties. This additional layer of security fortifies the federated learning process.

Moreover, federated learning facilitates the incorporation of differential privacy techniques. By guaranteeing that individual data samples remain private even during aggregate model updates, differential privacy ensures robust privacy preservation. This statistical privacy measure allows for efficient model training while upholding users’ privacy. Federated learning presents a promising approach for enhancing data privacy in IoT systems. By enabling decentralized training, preserving user privacy, incorporating encryption protocols, and embracing differential privacy techniques, federated learning empowers the development of secure and privacy-preserving machine learning models within IoT environments.

### 3.3. Federated Learning and Seamless Communication Integration for IoT Systems

The integration of federated learning and seamless communication in IoT systems offers significant potential to enhance data privacy, improve system efficiency, and facilitate collaborative learning. By combining these two approaches, IoT systems can harness the advantages of both federated learning and seamless communication to achieve heightened performance and security. Federated learning empowers IoT devices to train machine learning models locally, ensuring data privacy by keeping sensitive information within the devices themselves. It enables decentralized model training while upholding user privacy and data confidentiality. Meanwhile, seamless communication guarantees efficient and reliable data transmission between IoT devices and the central server, enabling real-time collaboration and model aggregation. 

Through the integration of seamless communication, IoT devices can securely exchange model updates and aggregated information with the central server. This necessitates the implementation of secure communication protocols, such as encryption and authentication mechanisms, to safeguard data during transit. Secure communication protocols like Transport Layer Security (TLS) can be deployed to encrypt data and establish secure connections, preserving the integrity and confidentiality of the transmitted information. The combination of federated learning and seamless communication facilitates continuous learning and adaptation within IoT systems. It enables devices to collectively train and enhance machine learning models without compromising data privacy. The federated learning approach ensures that each device contributes its local knowledge while safeguarding the privacy of individual data samples. Seamless communication ensures efficient model synchronization and aggregation, enabling devices to benefit from the collective intelligence of the entire IoT system. 

Moreover, this integration effectively addresses challenges stemming from the heterogeneity of IoT devices, encompassing variations in processing power and network connectivity. By optimizing communication protocols and adapting the learning process to accommodate different device capabilities, federated learning and seamless communication integration facilitates effective collaboration and knowledge sharing across diverse IoT devices.

### 3.4. Proposed Method

In order to protect against man-in-the-middle (MiM) attacks, the algorithm that we propose includes a signature creation procedure that includes a one-of-a-kind, one-time usability component. This component was developed expressly to defend against such attacks. (QSA) stands for “Quondam Signature Algorithm”, which is the name given to this particular technique. After a connection request has been made, the process for authenticating devices may then get underway. We use the date and time that is now displayed on the system to generate the one-time useable signature. A timestamp is created when the date from the system and the time that corresponds to it are combined. The timestamp vector is then multiplied by a substitution box (S), which is carried out for the purpose of encryption. It is important to note that the substitution box is organised in the form of a diagonal matrix with the dimensions 12 by 12. The Quondam Matrix (QM) with the dimensions 12 by 12 is the product that comes about as a consequence of this vector-substitution multiplication. The Quondam Signature (QS) is represented by the components of the Quondam Matrix that are diagonal to one another. The physical identification of the client, such as the MAC address, is added to the Quondam Signature (QS), and then it is sent to the server in order to enable the authentication of devices. This is performed in order to facilitate the authentication of devices.

**Figure 5:**
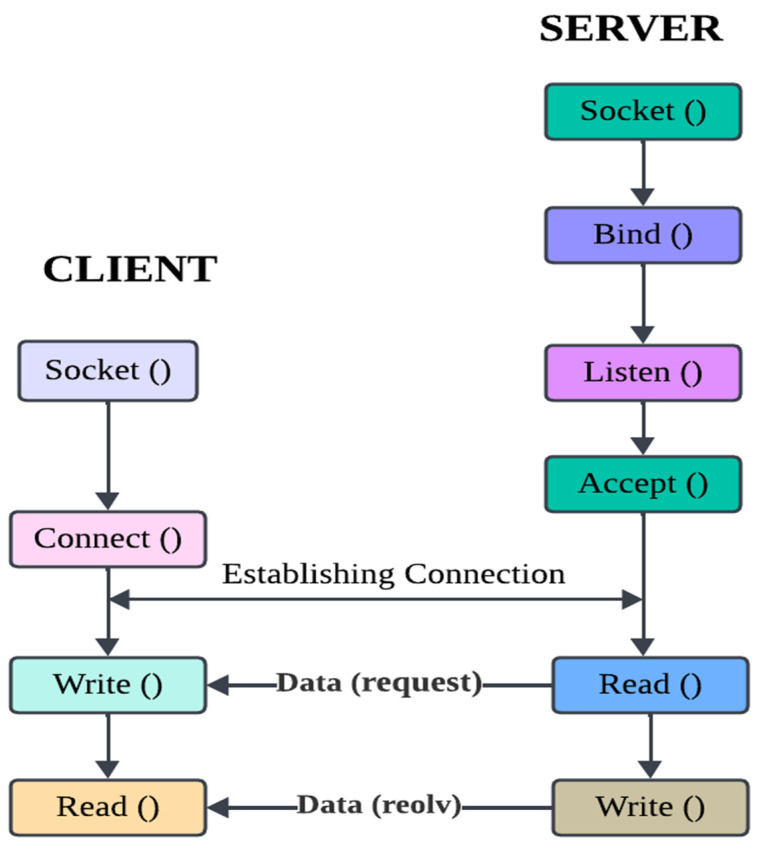
Process flow of socket programming application.

On their own PCs, authorised users will only find a “Substitution Box” and an algorithm that has already been pre-installed. A Personal Computer (PC), a NodeMCU, and a Real Time Clock (RTC) are the three main components that make up the experimental setup. The NodeMCU acts in the capacity of the server, and the PC plays the part of the client in this arrangement. The real-time clock is used in order to gather precise information on the system’s date and time. Figure 5 is a graphic representation of how the suggested system might really look in practise. In order to enable TCP/IP communication between the client (PC) and the server (NodeMCU), a socket programming application is built. This application is designed using the programming language C#. Figure 5 is a diagrammatic representation of the process flow of this socket programming application.

### 3.5. Proposed Algorithm

The method for the operation is broken down into two steps: first, the format setting, and then the signature creation.

The following is the configuration of formats using Algorithm 1.
**Algorithm 1**: Federated Quondam Signature Algorithm (FQSA)Step 1: StartStep 2:         Collect information from the client on the Client Connection Request (CR), Client Identity (CI), and Time Stamp (TS).Step 3:        Divide Client Identity (CI) into two parts:          - MAC ADD: Extract first eight digits of CI to obtain MAC address.          - Eight-Digit Hexadecimal: Convert MAC address to eight-digit hexadecimal value (each digit represents 4 bits).Step 4:        Initialize federated learning parameters:          - Define global model.          - Specify number of participating devices (N).          - Set communication rounds (R).Step 5:        For each communication round r in [1, R]:                For each participating device i in [1, N]:                          - Send global model to device i.                          - Device i updates local model using its own data and the received global model.                          - Device i trains its local model using federated learning.                          - Device i computes a local Quondam Signature (LQS) using the updated local model.Step 6:        Aggregate Local Quondam Signatures:          - Collect all local Quondam Signatures (LQS) from participating devices.          - Compute the global Quondam Signature (GQS) by aggregating the LQS.Step 7:        Perform Device Authentication:          - Attach GQS to the client’s connection request (CR).          - Send CR with GQS to the server for device authentication.Step 8:        End

The proposed method takes a comprehensive approach to address the real-time aspects of IoT (Internet of Things) by combining federated learning, the Quondam Signature Algorithm (QSA), and Physical Unclonable Functions (PUFs) in a way that ensures both security and efficiency while accommodating the time-sensitive nature of IoT applications.

Federated learning, a central component of the mechanism, is designed to accommodate real-time data processing in IoT environments. In federated learning, devices collaboratively train machine learning models without centrally pooling raw data. Instead, model updates are exchanged among devices, allowing local computations and updates to occur in real time. This enables IoT devices to learn and adapt continuously without the need to transfer large amounts of data to a central server, reducing latency and enhancing real-time responsiveness.

The Quondam Signature Algorithm (QSA) contributes to real-time security by offering a streamlined approach to data encryption, decryption, and authentication. The one-time accessible keys generated by the QSA are tailored to enhance authentication efficiency, a critical factor for IoT devices operating in real-time scenarios. By creating a unique signature for each interaction, the QSA contributes to swift and secure device authentication, minimizing delays and ensuring prompt responses to connection requests.

The incorporation of Physical Unclonable Functions (PUFs) further enriches real-time aspects. PUF-derived keys offer a means of ensuring device-specific authentication, which is crucial for maintaining the integrity of real-time interactions. The uniqueness and rapid retrieval of PUF-derived keys make them well-suited for IoT devices requiring swift and secure authentication within time-critical operations.

Collectively, the proposed mechanism leverages these components to address real-time aspects in IoT applications. Federated learning facilitates continuous learning and model updates without data centralization, aligning with the rapid pace of data generation and decision making in IoT environments. The QSA and PUFs enhance security and authentication efficiency, vital for maintaining real-time operations. By integrating these elements, the proposed mechanism presents a holistic approach that balances the demands of real-time IoT applications with the imperative of robust security and privacy measures.

- Set the current system’s date and time in a definite format as MM DD YYYY hh mm.
  - MM: Represents the two-digit month.
  - DD: Represents the two-digit day.
  - YYYY: Represents the four-digit year.
  - hh: Represents the two-digit hour.
  - mm: Represents the two-digit minute.

**Figure 6:**
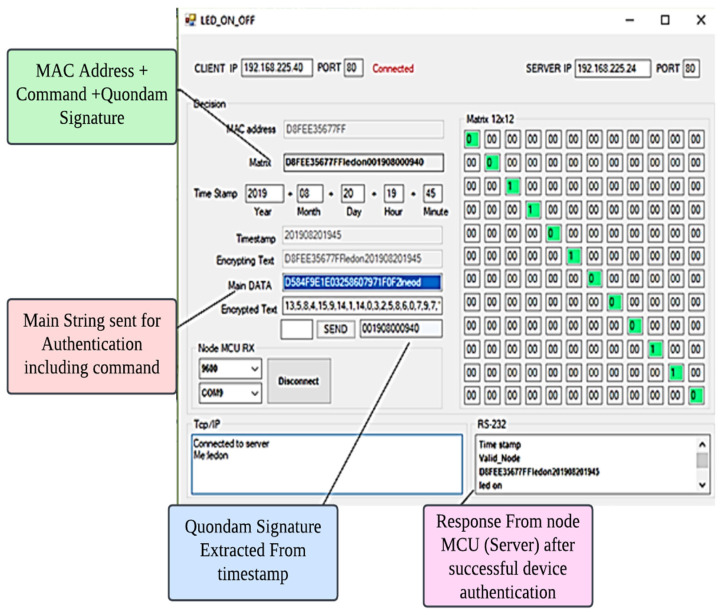
Implementation of proposed algorithm.

Convert the formatted date and time into a 12-digit character representation shown in Figure 6. 

### 3.6. Algorithm for Signature Generation

Step 1: To obtain the date and time of the system in the forms (D [] and (T []), respectively.

Step 2: Produce the time stamp vector TS [] by combining the current date and time of the system using the formula TS [] D [] + T [].

Step 3: Carry out the operation of multiplying the time stamp vector TS [] by the substitution matrix S [].

Step 4: Authorised users will need a pre-installed version of the replacement matrix S.

Step 5: The Quondam matrix QM is generated.

$$S{\left[\right]}_{12\times 12}=\begin{array}{cccccccccccc} S_{1} & 0 & 0 & 0 & 0 & 0 & 0 & 0 & 0 & 0 & 0 & 0\\ 0 & S_{2} & 0 & 0 & 0 & 0 & 0 & 0 & 0 & 0 & 0 & 0\\ 0 & 0 & S_{3} & 0 & 0 & 0 & 0 & 0 & 0 & 0 & 0 & 0\\ 0 & 0 & 0 & S_{4} & 0 & 0 & 0 & 0 & 0 & 0 & 0 & 0\\ 0 & 0 & 0 & 0 & S_{5} & 0 & 0 & 0 & 0 & 0 & 0 & 0\\ 0 & 0 & 0 & 0 & 0 & S_{6} & 0 & 0 & 0 & 0 & 0 & 0\\ 0 & 0 & 0 & 0 & 0 & 0 & S_{7} & 0 & 0 & 0 & 0 & 0\\ 0 & 0 & 0 & 0 & 0 & 0 & 0 & S_{8} & 0 & 0 & 0 & 0\\ 0 & 0 & 0 & 0 & 0 & 0 & 0 & 0 & S_{9} & 0 & 0 & 0\\ 0 & 0 & 0 & 0 & 0 & 0 & 0 & 0 & 0 & S_{10} & 0 & 0\\ 0 & 0 & 0 & 0 & 0 & 0 & 0 & 0 & 0 & 0 & S_{11} & 0\\ 0 & 0 & 0 & 0 & 0 & 0 & 0 & 0 & 0 & 0 & 0 & S_{12} \end{array}$$

where, S1, S2, ……, S12 ≠ 0.

Step 6: Compute the value of QM12 × 12 [ ].

Step 7: Extract the diagonal elements from QM12 × 12 [ ] to construct a 12-character-long QS [ ].

Step 8: Combine QS [ ] with CI [ ] and send the resulting data to the server as M20 × 1, where M20 × 1 = [M1 M2 M3…M20].

Step 9: The server receives the message M20 × 1 from the client.

Step 10: Encryption process concludes.

Step 11: The server segregates QS [ ] and CI [ ] from M20 × 1 as follows: M20 × 1 = QS [ ] 12 × 1 + CI [ ] 8 × 1.

Step 12: Formulate the Quondam signature as DM [ ] 12 × 12.

Step 13: Derive TS [ ] using the equation: TS [ ] = QM [ ] × S − 1.

Step 14: Decryption process concludes.

### 3.7. Federated Learning with PUF and QSA

The research paper introduces a holistic approach that synergizes the realms of federated learning, the Quondam Signature Algorithm (QSA), and the innovative utilization of Physical Unclonable Functions (PUFs), culminating in a robust and highly secure framework for modern information systems. At the heart of this amalgamation lies the role of PUFs, a groundbreaking concept that harnesses the inherent physical variations within electronic components to generate device-specific responses, known as PUF-derived keys. These keys are virtually impossible to replicate, forming the bedrock of enhanced security measures.

The strategic inclusion of PUFs in the proposed system encapsulates a multitude of advantages. Foremost among them is their instrumental role in elevating the device authentication process. By incorporating PUF-derived keys within the connection establishment phase, an additional layer of security is woven into the fabric of the communication channel. This intrinsic uniqueness of PUF responses ensures that each device has a distinct identity, mitigating the risk of unauthorized access and enhancing overall system integrity.

PUFs play a pivotal role in the intricate dance of the Quondam Signature Algorithm (QSA). These PUF-derived keys become the cornerstones for generating the one-time accessible keys, which are instrumental in the QSA’s data encryption, decryption, and authentication mechanisms. The robustness of the QSA is significantly fortified through the integration of PUFs, creating a dynamic synergy that amplifies the security of the system.

Notably, the implications of PUFs extend beyond the QSA. In the realm of federated learning, PUFs emerge as a crucial element in safeguarding data privacy. During the collaborative learning process, PUF-derived keys are harnessed to encrypt local updates before sharing them in federated rounds. This groundbreaking measure ensures that sensitive device data remain impervious to unauthorized access, even during the collaborative learning process, thereby championing the cause of privacy preservation.

The integration of Physical Unclonable Functions within the trifecta of federated learning, the Quondam Signature Algorithm, and PUFs encapsulates a paradigm shift in security and privacy considerations. It not only bolsters device authentication and data encryption but also fosters an environment where privacy remains paramount. The fusion of these cutting-edge concepts reflects a forward-looking approach to addressing the complex security challenges of modern information systems.

## 4. Result and Discussion

The Quondam Signature method was successfully validated with the help of the real-time IoT network. This system differentiates legal devices from malicious ones in an efficient manner, hence restricting access granted to unauthorized users. The process of implementation also included the step of estimating the expenses of communication. With the method that we have suggested, the amount of bits needed for successful transmission varies from 192 to 220.

The experimental setup for evaluating the Quondam Signature Algorithm within a real-time IoT network was meticulously designed to ascertain its efficacy. The architecture encompassed a diversified array of IoT devices, each playing distinct roles within the network. IoT sensors, actuators, gateways, and intermediary nodes were intricately interconnected using a predefined network topology, fostering interactions akin to real-world IoT scenarios. Communication was facilitated through the MQTT protocol, with data rates and frequencies tailored to emulate real-time data transmission and event-triggered communication.

The experiment encompassed a series of scenarios reflecting authentic use cases within IoT environments. Devices were meticulously configured, with hardware specifications encompassing computational power, memory, and pertinent components. The Quondam Signature Algorithm was methodically implemented on these devices, involving the intricate interplay of signature generation, verification, and the integration of Physical Unclonable Functions (PUFs) and federated learning strategies where relevant.

Authentication processes were instrumental in distinguishing between genuine and malicious devices, enabling the algorithm to exercise access control mechanisms with finesse. Communication cost, a critical metric, was rigorously calculated by capturing and analysing communication logs. The proposed approach demonstrated a communication cost range of 192–220 bits, further underlining its efficiency in real-time IoT scenarios.

In the evaluation phase, an array of metrics, including authentication accuracy, false positives/negatives, and communication overhead, were scrutinized to gauge the performance of the Quondam Signature Algorithm. The resulting metrics validated the algorithm’s ability to effectively authenticate devices in real-time, bolstering the security posture of IoT networks. The meticulously devised experimental setup, meticulous implementation, and comprehensive evaluation collectively underscored the algorithm’s prowess in addressing real-time demands while fortifying IoT security.

The IoT architecture employed in the experimental setup was meticulously designed to emulate a practical and diverse network scenario. A total of 50 IoT devices were strategically distributed across the network, encompassing a mix of IoT sensors, actuators, and gateways. This heterogeneous ensemble allowed for the simulation of complex interactions within the IoT ecosystem.

In terms of communication parameters, the devices operated on a combination of data rates and frequencies tailored to mirror real-world IoT dynamics. Data transmission intervals were set at 5 s intervals for sensor devices, facilitating periodic updates. Meanwhile, event-triggered communication was initiated by actuators in response to predefined thresholds, with real-time responsiveness being a primary focus. Communication utilized the MQTT protocol, known for its lightweight and publish-subscribe architecture, thus aligning with the resource constraints often encountered in IoT devices.

This intricate IoT architecture, housing a diverse array of devices, alongside communication parameters optimized for real-time interactions, laid the foundation for a robust experimental framework. The setup was carefully designed to not only reflect the complexities of real-world IoT deployments but also to ensure that the proposed Quondam Signature Algorithm could effectively operate within these dynamic and challenging scenarios.

The proposed system’s performance is evaluated in terms of computational cost and energy consumption, i.e., carried out while performing operations such as key generation, signing, and verification, shown in Table 2. The offline signature scheme uses the Inversion Operation (IO) and the Pairing Operation (PO). The IO hides the inverted values in the Oracle outputs. The PO is also known by the name bilinear mapping, which is mainly used to design complex cryptographic protocols. The PO mainly generates short signatures, which can be further derived by performing scalar multiplication on the elliptical curve. The offline scheme uses scalar multiplication (SM) for the hash function (H) generated during the G1 execution. SM is mainly the multiplication of a point Q present in an elliptical curve over the finite field. It is like multiplying any point by its scalar value. A point Q is chosen from a set of rational points in a given elliptical curve, and the value is incremented by adding the intermediate result to it. The SM serves as a significant concept in Elliptic Curve Cryptography (ECC), which uses a binary validation method. If the result is a bit value, ‘0’ means a point-doubling operation is performed. If the result is of a bit value, ‘1’ means point addition with doubling is performed when the attacker can differentiate between the point-doubling and point-addition operation, the message’s secret.

### Computational Time Taken by the Different Algorithms 

**Figure 7:**
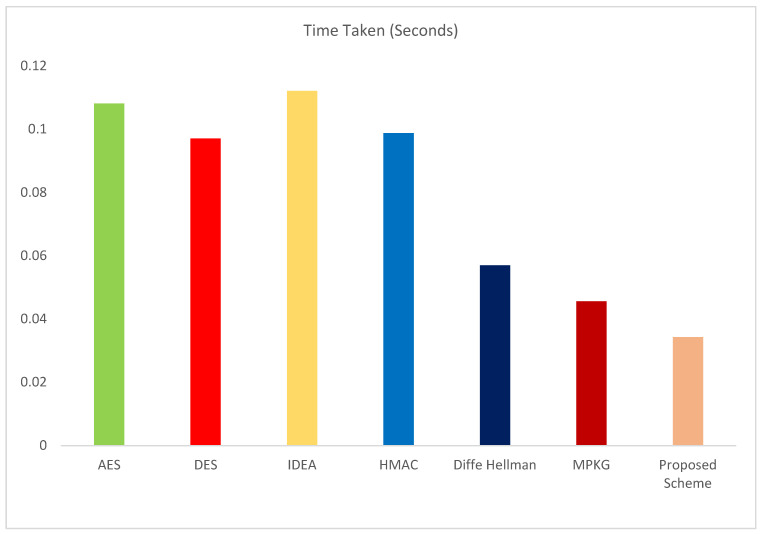
Computational time comparison of different schemes.

Table 3 summarizes the proposed scheme’s execution on energy consumption is less than a bit joule, making the scheme an energy-efficient one. A degree of temporal complexity is introduced by the signature schemes in order to confirm the authenticity and integrity of messages that are sent back and forth between nodes. The temporal complexity, on the other hand, is greatly reduced by the plan that was suggested. In addition, the performance advantages that ECC has over RSA become more evident as the key size grows, particularly in terms of the amount of time spent on execution and the amount of energy used. The results of the comparison between the proposed system and earlier sensor network designs are shown in Table 4. These results demonstrate that the suggested scheme is superior shown in Figure 7. 

**Figure 8:**
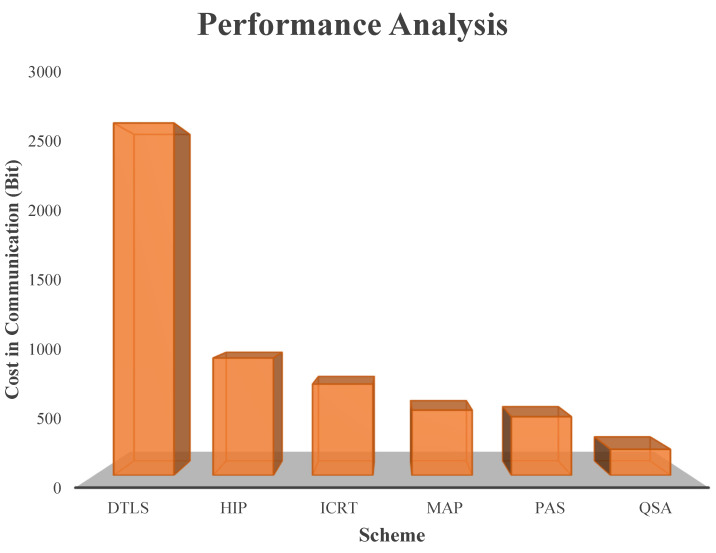
Cost in communication during authentication.

The time complexities of various schemes are evaluated and compared with each other, as shown in Table 4 and Figure 8.

Compared to the other schemes, the proposed method’s time complexity is mainly reduced by doing complex computations in the offline phase and generating the online phase’s signature. The elliptic curve digital signature algorithm based online, an offline signature scheme used in the proposed scheme reduces the time during key generation, signing, and verification, which differentiates the proposed scheme from others. The SBM scheme is the slowest of all the schemes described above. The above results demonstrate the time complexity of more than 25 rounds, including a 1500 signature generation and 1500 verifications. The main advantage of identity-based online/offline schemes is reducing the signature size and computational overhead during signature generation. The proposed scheme provides the same size for both the public key and the user’s signature. The key generated for the ID-based online/offline scheme is 20 bytes (160 bits).

## 5. Conclusions and Future Scope

In order to combat the problem of random assaults on IoT systems, this research proposes cutting-edge data encryption and decryption techniques. The suggested techniques successfully reduce the danger of man-in-the-middle attacks by creating device signatures that are only accessible once using the Quondam Signature Algorithm (QSA). The findings show how effective the algorithms are at lowering communication costs, expressed in terms of bits transferred, and they also show the possibility for further optimization. The research emphasizes the significance of the findings by contrasting alternative communication cost strategies. The suggested remedy can also be expanded to cover other common assaults on IoT systems. In order to meet the demands of smartphones and other IoT devices, it provides a secure command protocol with programmable settings, assuring a high level of security.

There is the potential for more research in the future about the possibility of decreasing the length of device signatures that are used in the process of authenticating devices. Investigating a variety of abbreviated physical identities, such as CPU signatures, co-processor IDs, and memory unit signatures, is one such approach that might be taken. In addition, there is a possibility for extensive study into the compression of created timestamps, with the goal of achieving an even higher decrease in the costs associated with communication.

## Figures and Tables

**Table 1:** Summary of standard cryptography for location information [35].

Scheme	Configuration	Authority	Integration	Shared Secret ^1^
Check Sum			Yes	
Encryption ^2^ + Signature ^2^	Yes	Yes	Yes	
Digital Signature ^2^		Yes	Yes	
Authenticated Encryption	Yes	Yes	Yes	Yes
Message Authentication		Yes	Yes	Yes
Secret-Key Encryption	Yes	Yes	Yes	Yes
Public-Key Encryption ^2^	Yes		Yes	

^1^ shared key generated using user input and random number, ^2^ it I used for authentication process.

**Table 2:** Comparison of evaluation cost as well as the key size.

Methods	[22]	[23]	[24]	[25]	[26]	[27]	Proposed Scheme
Offline evaluation	3MP + 1M M	3MP + 1 MM	3MP + 1 MM	3MP + 1 MM	3MP + 1 MM	3MP + 1 MM	3MP + 1 MM
Online evaluation	3A + 5M	2A + 5M	2A + 4M	2A + 3M	A + 3M	3M	2M
Offline storage	2624	5056	2624	5056	2624	3632	1312
Ciphertext length	2144	6464	2144	4320	3424	2144	1280
No. of pairing for decryption	8	7	5	5	4	4	2

**Table 3:** Computational cost calculated for various schemes.

Scheme	Parameter Generation			Signing			Verification			
	SM	H	PO	SM	IO	H	PO	SM	IO	H
AES [28]	1	0	0	1	0	0	0	2	0	1
DES [29]	1	0	2	1	1	1	0	4	1	1
IDEA [30]	1	0	0	1	1	1	1	1	0	1
HMAC [31]	1	0	0	1	1	1	1	0	1	1
Diffe Hellman [32]	1	0	2	1	1	1	0	2	0	1
MPKG [33]	1	1	0	1	1	1	1	1	0	1
Proposed Scheme	1	1	0	0	1	1	1	1	1	1

Note: Inversion Operation (IO), Pairing Operation (PO), scalar multiplication (SM) for the hash function (H).

**Table 4:** Execution time of different schemes.

Scheme	Time Taken (Seconds)
AES	0.1081002
DES	0.0970700
IDEA	0.1121040
HMAC	0.0987641
Diffe Hellman	0.0570001
MPKG	0.0456002
Proposed Scheme	0.0342760

## Data Availability

Data were provided on the request.

## References

[1] Iqbal W., Abbas H., Daneshmand M., Rauf B., Bangash Y.A. (2020). An In-Depth Analysis of IoT Security Requirements, Challenges, and Their Countermeasures via Software-Defined Security. IEEE Internet Things J..

[2] Karie N.M., Sahri N.M., Yang W., Valli C., Kebande V.R. (2021). A Review of Security Standards and Frameworks for IoT-Based Smart Environments. IEEE Access.

[3] Lam K.-Y., Mitra S., Gondesen F., Yi X. (2022). ANT-Centric IoT Security Reference Architecture—Security-by-Design for Satellite-Enabled Smart Cities. IEEE Internet Things J..

[4] Chen F., Luo D., Li J., Leung V.C.M., Li S., Fan J. (2023). Arm PSA-Certified IoT Chip Security: A Case Study. Tsinghua Sci. Technol..

[5] Verma S., Kawamoto Y., Kato N. (2021). A Network-Aware Internet-Wide Scan for Security Maximization of IPv6-Enabled WLAN IoT Devices. IEEE Internet Things J..

[6] Nosouhi M.R., Sood K., Grobler M., Doss R. (2022). Towards Spoofing Resistant Next Generation IoT Networks. IEEE Trans. Inf. Forensics Secur..

[7] Zhou W., Cao C., Huo D., Cheng K., Zhang L., Guan L., Liu T., Jia Y., Zheng Y., Zhang Y. (2021). Reviewing IoT Security via Logic Bugs in IoT Platforms and Systems. IEEE Internet Things J..

[8] You M., Kim Y., Kim J., Seo M., Son S., Shin S., Lee S. (2022). FuzzDocs: An Automated Security Evaluation Framework for IoT. IEEE Access.

[9] Bouzidi M., Gupta N., Cheikh F.A., Shalaginov A., Derawi M. (2022). A Novel Architectural Framework on IoT Ecosystem, Security Aspects and Mechanisms: A Comprehensive Survey. IEEE Access.

[10] Al-Garadi M.A., Mohamed A., Al-Ali A.K., Du X., Ali I., Guizani M. (2020). A Survey of Machine and Deep Learning Methods for Internet of Things (IoT) Security. IEEE Commun. Surv. Tutor..

[11] Hussain F., Hussain R., Hassan S.A., Hossain E. (2020). Machine Learning in IoT Security: Current Solutions and Future Challenges. IEEE Commun. Surv. Tutor..

[12] Jung J., Kim B., Cho J., Lee B. (2022). A Secure Platform Model Based on ARM Platform Security Architecture for IoT Devices. IEEE Internet Things J..

[13] Samaila M.G., Sequeiros J.B.F., Simoes T., Freire M.M., Inacio P.R.M. (2020). IoT-HarPSecA: A Framework and Roadmap for Secure Design and Development of Devices and Applications in the IoT Space. IEEE Access.

[14] Zhang J., Wang Y., Li S., Shi S. (2021). An Architecture for IoT-Enabled Smart Transportation Security System: A Geospatial Approach. IEEE Internet Things J..

[15] Liao B., Ali Y., Nazir S., He L., Khan H.U. (2020). Security Analysis of IoT Devices by Using Mobile Computing: A Systematic Literature Review. IEEE Access.

[16] Wang J., Hao S., Wen R., Zhang B., Zhang L., Hu H., Lu R. (2021). IoT-Praetor: Undesired Behaviors Detection for IoT Devices. IEEE Internet Things J..

[17] Ashok K., Gopikrishnan S. (2023). Statistical Analysis of Remote Health Monitoring Based IoT Security Models & Deployments from a Pragmatic Perspective. IEEE Access.

[18] Kim J., Astillo P.V., Sharma V., Guizani N., You I. (2022). MoTH: Mobile Terminal Handover Security Protocol for HUB Switching Based on 5G and Beyond (5 GB) P2MP Backhaul Environment. IEEE Internet Things J..

[19] Lounis K., Zulkernine M. (2020). Attacks and Defenses in Short-Range Wireless Technologies for IoT. IEEE Access.

[20] Karmakar K.K., Varadharajan V., Nepal S., Tupakula U. (2021). SDN-Enabled Secure IoT Architecture. IEEE Internet Things J..

[21] Cook J., Rehman S.U., Khan M.A. (2023). Security and Privacy for Low Power IoT Devices on 5G and Beyond Networks: Challenges and Future Directions. IEEE Access.

[22] Swamy S.N., Kota S.R. (2020). An Empirical Study on System Level Aspects of Internet of Things (IoT). IEEE Access.

[23] Ren J., Li J., Liu H., Qin T. (2022). Task offloading strategy with emergency handling and blockchain security in SDN-empowered and fog-assisted healthcare IoT. Tsinghua Sci. Technol..

[24] Li H., Yu S., Feng W., Chen Y., Zhang J., Qin Z., Zhu Z., Wozniak M. (2023). Exploiting Dynamic Vector-Level Operations and a 2D-Enhanced Logistic Modular Map for Efficient Chaotic Image Encryption. Entropy.

[25] Wen H., Kang S., Wu Z., Lin Y., Huang Y. (2023). Dynamic RNA Coding Color Image Cipher Based on Chain Feedback Structure. Mathematics.

[26] Ali A., Al-Rimy B.A.S., Alsubaei F.S., Almazroi A.A., Almazroi A.A. (2023). HealthLock: Blockchain-Based Privacy Preservation Using Homomorphic Encryption in Internet of Things Healthcare Applications. Sensors.

[27] Zhang J., Li G., Marshall A., Hu A., Hanzo L. (2020). A New Frontier for IoT Security Emerging from Three Decades of Key Generation Relying on Wireless Channels. IEEE Access.

[28] Lins F.A.A., Vieira M. (2021). Security Requirements and Solutions for IoT Gateways: A Comprehensive Study. IEEE Internet Things J..

[29] Fernandez-Carames T.M. (2020). From Pre-Quantum to Post-Quantum IoT Security: A Survey on Quantum-Resistant Cryptosystems for the Internet of Things. IEEE Internet Things J..

[30] Zhang J., Zhong H., Cui J., Xu Y., Liu L. (2021). SMAKA: Secure Many-to-Many Authentication and Key Agreement Scheme for Vehicular Networks. IEEE Trans. Inf. Forensics Secur..

[31] Khurshid A., Alsaaidi R., Aslam M., Raza S. (2022). EU Cybersecurity Act and IoT Certification: Landscape, Perspective and a Proposed Template Scheme. IEEE Access.

[32] Sood K., Karmakar K.K., Yu S., Varadharajan V., Pokhrel S.R., Xiang Y. (2020). Alleviating Heterogeneity in SDN-IoT Networks to Maintain QoS and Enhance Security. IEEE Internet Things J..

[33] Kornaros G. (2022). Hardware-Assisted Machine Learning in Resource-Constrained IoT Environments for Security: Review and Future Prospective. IEEE Access.

[34] Rathee G., Ahmad F., Jaglan N., Konstantinou C. (2023). A Secure and Trusted Mechanism for Industrial IoT Network Using Blockchain. IEEE Trans. Ind. Inform..

[35] Vangala A., Das A.K., Mitra A., Das S.K., Park Y. (2023). Blockchain-Enabled Authenticated Key Agreement Scheme for Mobile Vehicles-Assisted Precision Agricultural IoT Networks. IEEE Trans. Inf. Forensics Secur..

[36] Zhang X., Zhong H., Fan C., Bolodurina I., Cui J. (2022). CBACS: A Privacy-Preserving and Efficient Cache-Based Access Control Scheme for Software Defined Vehicular Networks. IEEE Trans. Inf. Forensics Secur..

[37] Iqbal W., Abbas H., Deng P., Wan J., Rauf B., Abbas Y., Rashid I. (2021). ALAM: Anonymous Lightweight Authentication Mechanism for SDN-Enabled Smart Homes. IEEE Internet Things J..

[38] Breitenbacher D., Homoliak I., Aung Y.L., Elovici Y., Tippenhauer N.O. (2022). HADES-IoT: A Practical and Effective Host-Based Anomaly Detection System for IoT Devices (Extended Version). IEEE Internet Things J..

[39] Cui J., Wang F., Zhang Q., Xu Y., Zhong H. (2021). Anonymous Message Authentication Scheme for Semitrusted Edge-Enabled IIoT. IEEE Trans. Ind. Electron..

[40] Xenofontos C., Zografopoulos I., Konstantinou C., Jolfaei A., Khan M.K., Choo K.-K.R. (2022). Consumer, Commercial, and Industrial IoT (In)Security: Attack Taxonomy and Case Studies. IEEE Internet Things J..

[41] Shao Z., Weng J., Zhang Y., Wu Y., Li M., Weng J., Luo W., Yu S. (2022). Peripheral-Free Device Pairing by Randomly Switching Power. IEEE Trans. Dependable Secur. Comput..

[42] Khan M.N., Rao A., Camtepe S. (2021). Lightweight Cryptographic Protocols for IoT-Constrained Devices: A Survey. IEEE Internet Things J..

[43] Sharma V., You I., Andersson K., Palmieri F., Rehmani M.H., Lim J. (2020). Security, Privacy and Trust for Smart Mobile- Internet of Things (M-IoT): A Survey. IEEE Access.

[44] Adarbah H.Y., Moghadam M.F., Maata R.L.R., Mohajerzadeh A., Al-Badi A.H. (2023). Security Challenges of Selective Forwarding Attack and design a Secure ECDH-Based Authentication Protocol to Improve RPL Security. IEEE Access.

[45] Amato F., Casola V., Cozzolino G., De Benedictis A., Moscato F. (2020). Exploiting Workflow Languages and Semantics for Validation of Security Policies in IoT Composite Services. IEEE Internet Things J..

[46] Allifah N.M., Zualkernan I.A. (2022). Ranking Security of IoT-Based Smart Home Consumer Devices. IEEE Access.

[47] Wazid M., Das A.K., Shetty S. (2022). TACAS-IoT: Trust Aggregation Certificate-Based Authentication Scheme for Edge-Enabled IoT Systems. IEEE Internet Things J..

[48] Zarca A.M., Bernabe J.B., Skarmeta A., Calero J.M.A. (2020). Virtual IoT HoneyNets to Mitigate Cyberattacks in SDN/NFV-Enabled IoT Networks. IEEE J. Sel. Areas Commun..

[49] Ilyas B., Raouf S.M., Abdelkader S., Camel T., Said S., Lei H. (2022). An Efficient and Reliable Chaos-Based IoT Security Core for UDP/IP Wireless Communication. IEEE Access.

[50] Ashraf I., Park Y., Hur S., Kim S.W., Alroobaea R., Bin Zikria Y., Nosheen S. (2023). A Survey on Cyber Security Threats in IoT-Enabled Maritime Industry. IEEE Trans. Intell. Transp. Syst..

[51] Park C.-S., Nam H.-M. (2020). Security Architecture and Protocols for Secure MQTT-SN. IEEE Access.

[52] Nabeel N., Habaebi M.H., Islam M.D.R. (2021). Security Analysis of LNMNT-LightWeight Crypto Hash Function for IoT. IEEE Access.

[53] Zhang P., Wang Y., Kumar N., Jiang C., Shi G. (2022). A Security- and Privacy-Preserving Approach Based on Data Disturbance for Collaborative Edge Computing in Social IoT Systems. IEEE Trans. Comput. Soc. Syst..

[54] Bera B., Das A.K., Garg S., Piran J., Hossain M.S. (2022). Access Control Protocol for Battlefield Surveillance in Drone-Assisted IoT Environment. IEEE Internet Things J..

[55] Oh M.-K., Lee S., Kang Y., Choi D. (2020). Wireless Transceiver Aided Run-Time Secret Key Extraction for IoT Device Security. IEEE Trans. Consum. Electron..

[56] Cui J., Bian F., Zhong H., Zhang Q., Xu S., Gu C., Liu L. (2022). An Anonymous and Outsourcing-Supported Multiauthority Access Control Scheme with Revocation for Edge-Enabled IIoT System. IEEE Syst. J..

[57] Krishnan P., Jain K., Buyya R., Vijayakumar P., Nayyar A., Bilal M., Song H. (2022). MUD-Based Behavioral Profiling Security Framework for Software-Defined IoT Networks. IEEE Internet Things J..

[58] Khedr W.I., Gouda A.E., Mohamed E.R. (2023). FMDADM: A Multi-Layer DDoS Attack Detection and Mitigation Framework Using Machine Learning for Stateful SDN-Based IoT Networks. IEEE Access.

[59] Srinivas J., Das A.K., Wazid M., Vasilakos A.V. (2021). Designing Secure User Authentication Protocol for Big Data Collection in IoT-Based Intelligent Transportation System. IEEE Internet Things J..

[60] Wang Z., Liu D., Sun Y., Pang X., Sun P., Lin F., Lui J.C.S., Ren K. (2022). A Survey on IoT-Enabled Home Automation Systems: Attacks and Defenses. IEEE Commun. Surv. Tutor..

[61] Liu C., Zhang Y., Xu J., Zhao J., Xiang S. (2022). Ensuring the Security and Performance of IoT Communication by Improving Encryption and Decryption with the Lightweight Cipher uBlock. IEEE Syst. J..

[62] Lee E., Seo Y.-D., Oh S.-R., Kim Y.-G. (2021). A Survey on Standards for Interoperability and Security in the Internet of Things. IEEE Commun. Surv. Tutor..

[63] He X., Wang J., Liu J., Ding W., Han Z., Wang B., Nebhen J., Wang W. (2023). DNS Rebinding Threat Modeling and Security Analysis for Local Area Network of Maritime Transportation Systems. IEEE Trans. Intell. Transp. Syst..

[64] Perez S., Hernandez-Ramos J.L., Raza S., Skarmeta A. (2020). Application Layer Key Establishment for End-to-End Security in IoT. IEEE Internet Things J..

[65] Cui J., Ouyang F., Ying Z., Wei L., Zhong H. (2022). Secure and Efficient Data Sharing Among Vehicles Based on Consortium Blockchain. IEEE Trans. Intell. Transp. Syst..

[66] Bagaa M., Taleb T., Bernabe J.B., Skarmeta A. (2020). A Machine Learning Security Framework for Iot Systems. IEEE Access.

[67] Hatcher W.G., Qian C., Liang F., Liao W., Blasch E.P., Yu W. (2022). Secure IoT Search Engine: Survey, Challenges Issues, Case Study, and Future Research Direction. IEEE Internet Things J..

[68] Aldahmani A., Ouni B., Lestable T., Debbah M. (2023). Cyber-Security of Embedded IoTs in Smart Homes: Challenges, Requirements, Countermeasures, and Trends. IEEE Open J. Veh. Technol..

[69] Dushku E., Rabbani M., Conti M., Mancini L.V., Ranise S. (2020). SARA: Secure Asynchronous Remote Attestation for IoT Systems. IEEE Trans. Inf. Forensics Secur..

[70] Shafiq M., Tian Z., Bashir A.K., Du X., Guizani M. (2021). CorrAUC: A Malicious Bot-IoT Traffic Detection Method in IoT Network Using Machine-Learning Techniques. IEEE Internet Things J..

